# Erythema Multiforme Major following SARS‐CoV‐2 vaccine

**DOI:** 10.1002/ccr3.4947

**Published:** 2021-10-13

**Authors:** Alberto Maria Saibene, Andrea Alliata, Anna Teresa Cozzi, Alice Ottavi, Sofia Spagnolini, Carlotta Pipolo, Alberto Maccari, Giovanni Felisati

**Affiliations:** ^1^ Otolaryngology Unit Santi Paolo e Carlo Hospital Department of Health Sciences Università degli Studi di Milano Milan Italy; ^2^ Department of Health Sciences Università degli Studi di Milano Milan Italy

**Keywords:** adverse drug reactions, Coronavirus infections, COVID‐19, oral disease, vaccine

## Abstract

Erythema multiforme major, an immune‐mediated skin reaction to infections or medications with oral involvement, should be taken into account as a potential side effect of several vaccines, including SARS‐CoV‐2. Correct patient history collection allows prompt recognition and subsequent successful medical management with oral corticosteroids.

## Graphical abstract

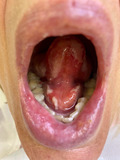

## 1 INTRODUCTION

Erythema multiforme (EM) is an immune‐mediated skin reaction to infections or medications. Its oral cavity and mucosae‐involving presentation (erythema multiforme major, EMM) represent a serious, occasionally life‐threatening condition.^1^

EM may represent an adverse drug reaction (ADR) following vaccine administration,^2^ and it has been recently shown to sporadically follow SARS‐CoV‐2 vaccination.^3^ No EMM case, especially with prevalent oral involvement, has ever been documented to date following COVID‐19 vaccines.

We report a case of a 58‐year‐old woman with oral and skin lesions due to SARS‐CoV‐2 vaccine‐induced EMM.

## 2 REPORT OF A CASE

A 58‐year‐old woman presented to our emergency department reporting oral lesions, oral floor swelling, excruciating oral burning pain, dysphagia and odynophagia, and nocturnal fever the day after receiving the second dose of mRNA‐1273 SARS‐CoV‐2 vaccine (Moderna Inc). She also reported cutaneous lesions on the right thigh and calf. She didn't report any ADR after the first dose, while she did recall an analogous clinical picture after administration of metamizole and penicillin (respectively 27 and 16 years before). The patient was under treatment with oral sertraline, lorazepam, and atorvastatin, and she didn't take any other drug during the previous month.

The patient presented to our emergency department on day 5 after the appearance of lesions, due to worsening of the clinical picture and the inability to hydrate and feed.

**FIGURE 1:**
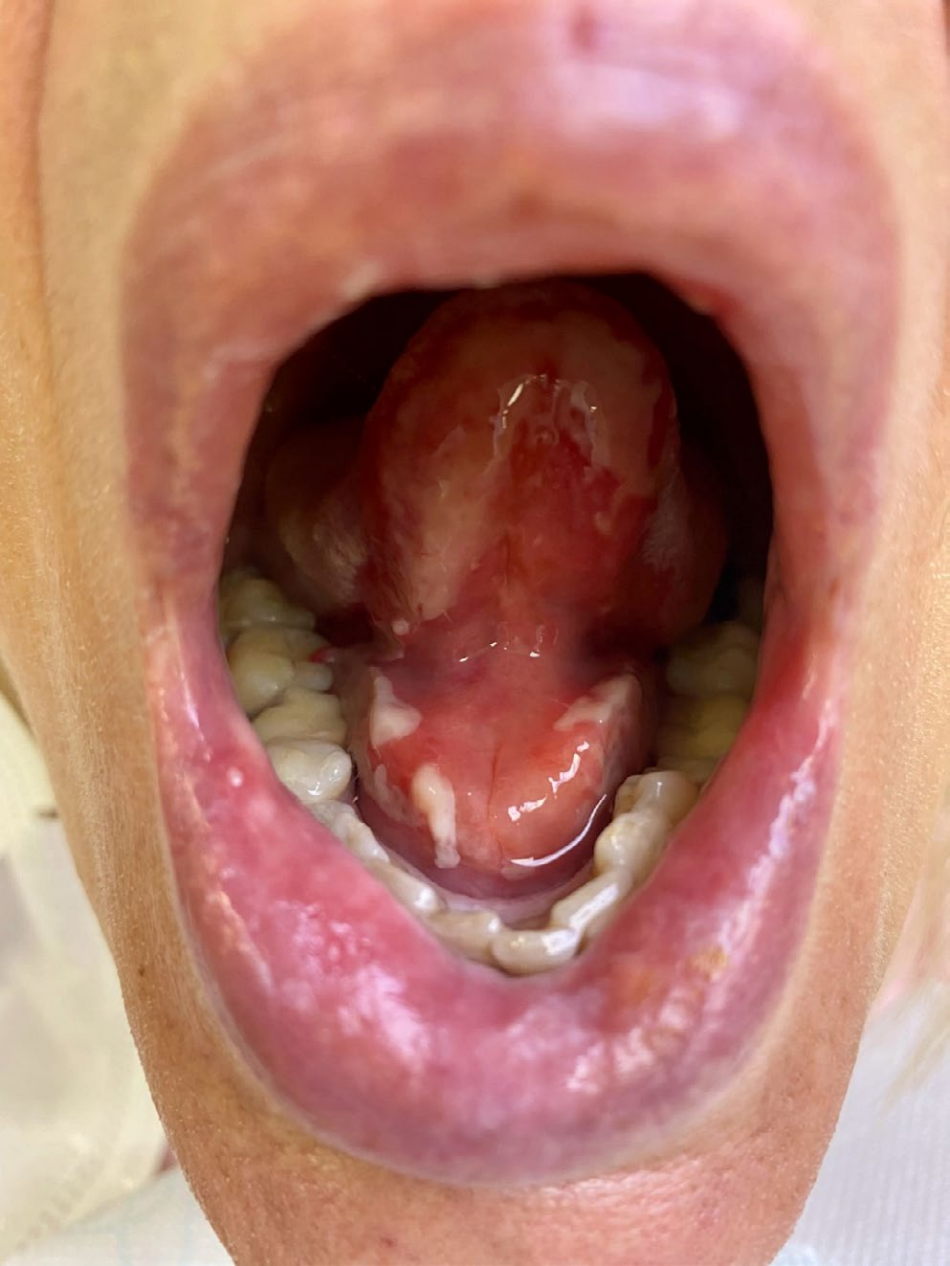
Oral examination on the day of admission showing oral floor swelling accompanied by diffuse oral mucosal erosive lesions, without signs of bacterial or fungal infection

**FIGURE 2:**
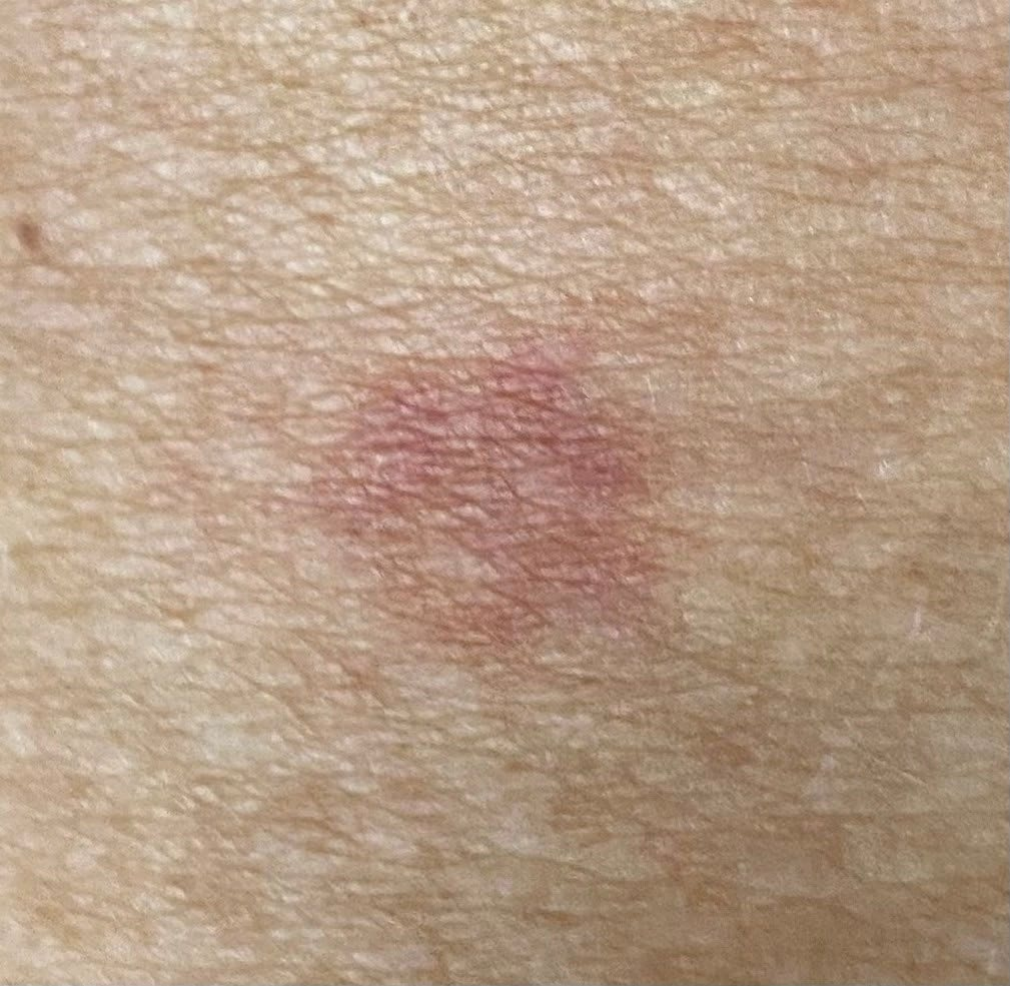
Right tight round erythematosus‐brownish target‐like lesions as appearing on the day of admission

The otolaryngological evaluation confirmed an oral floor swelling accompanied by diffuse oral mucosal erosive lesions (Figure 1). The dermatologist identified two round erythematosus‐brownish target‐like lesions (on the right thigh and calf) on unscathed skin, roughly 1 cm wide, without bubbles or detachment (Figure 2). There was no conjunctivitis or genital involvement. Laboratory tests identified only a moderate C‐reactive protein increase (23.9 mg/L, normal range <10 mg/L) and the nasopharyngeal swab for SARS‐CoV‐2 proved negative. The infectious disease specialist requested serological testing for Chlamydia pneumoniae (IgG and IgM), Mycoplasma pneumoniae (IgG and IgM), T. pallidum, herpesviruses 1 and 2, hepatitis C, and hepatitis B. All serological testing was normal and safe for herpesvirus IgG (22.1 titration index, with positive values >1) and HBsAb resulting from mandatory vaccination (438 international units per liter, with positive values >10). Based on clinical appearance and medical history, a diagnosis of drug‐related erythema multiforme was made,^1^ and IV methylprednisolone 1 mg/kg and fluid supplementation were started. IV morphine was required for pain management during the first 48 hours. A progressive improvement of the clinical picture with C‐reactive protein normalization (9.3 mg/L) allowed for oral fluid intake on day 4, while oral feeding and IV steroids tapering began on day 5. The following day, the patient was dismissed on oral prednisone tapering, with almost complete resolution of the clinical picture. No recurrence was noted at the 1‐month evaluation.

## 3 DISCUSSION

To our knowledge, the authors present the first mRNA‐vaccine‐induced case of EMM. Although the relationship between EM and immunization procedures is already established, its link with recent mRNA vaccines is less clear. The pathophysiological mechanics might mimic the same immune response to SARS‐CoV‐2‐mediated EM.^3^

This report illustrates the diagnostic challenge posed by EMM, ie, its prevalently oral manifestation. In contrast with full‐scale dermatological presentations (predominant both in mild EM cases, Stevens‐Johnson syndrome, or toxic epidermal necrolysis—the latter two already described after SARS‐CoV‐2 immunization^4^), oral lesions and symptoms might be also suggestive of fungal or viral infections. Mycoplasma pneumoniae‐induced rash and mucositis might share the presentation with EMM, although often in younger patients, usually in their first or second decade.^5^ In the presented case, serological information allowed to rule out M. pneumoniae infection. It has to be noted also that the differential diagnosis between Stevens‐Johnson syndrome and EMM might represent a clinical issue.^6^ Histological evaluation of skin lesions, which bear different features in EMM and Stevens‐Johnson syndrome might represent an additional tool. In this specific case, the dermatologist considered the EMM diagnosis consistent with the clinical features and therefore decided not to perform any biopsy on the few skin lesions that appeared on the patient.

Dermatological manifestations can be mild or subtle and, unless the patient's drug and personal history hint at an immune‐mediated reaction, the corticosteroid treatment might get delayed.

Although EMM is a known vaccine ADR, the exceptional pandemic circumstances turned the spotlight back on potential vaccine risks, which have been long known and adequately managed in a large number of patients.^7^, ^8^ ADRs to new mRNA vaccines have been therefore widely covered by the media and often speculatively manipulated into sensational claims, impacting negatively on vaccine public acceptance.^9^, ^10^ The prompt recognition of infrequent ADRs such as EMM represents the most suitable tool to improve patients' outcomes and trust toward healthcare providers.

## CONFLICT OF INTEREST

None declared.

## AUTHOR CONTRIBUTIONS

Alberto Maria Saibene and Andrea Alliata finalized the paper and wrote the final version. Anna Teresa Cozzi, Alice Ottavi, and Sofia Spagnolini drafted the paper. Carlotta Pipolo performed the clinical evaluations and prepared the images. Alberto Maccari and Giovanni Felisati acted as senior consultants for this paper.

## CONSENT

The authors confirm that patient consent has been signed and collected in accordance with the journal's patient consent policy.

## Data Availability

Data sharing not applicable to this article as no datasets were generated or analysed during the current study.
